# Expression of COL6A1 in Tumor Matrix of Cholangiocarcinoma and Its Mechanism of Tumor Progression

**DOI:** 10.1002/cnr2.70716

**Published:** 2026-10-09

**Authors:** Weibin Zhang, Chongshi Gao, Chongzhou Lan, Jia Dong, Boxing Xu, Yingmei Li, Jin Dai, Baitong Che, Cheng Li, Gang Dong, Yunfei Wu, Bo Li, Cheng Yang, Lingmei Kong

**Affiliations:** ^1^ Jinzhou Central Hospital Jinzhou Liaoning China; ^2^ Graduate School, Jinzhou Medical University Postgraduate Training Base (Jinzhou Central Hospital) Jinzhou Liaoning China; ^3^ Department of Radiology Jinzhou Maternal and Infant Hospital Jinzhou Liaoning China

**Keywords:** cholangiocarcinoma, COL6A1, molecular mechanisms, prognostic markers, tumor progression, tumor stroma

## Abstract

**Background:**

Cholangiocarcinoma (CCA), a malignant tumor originating from the bile duct epithelial cells, is characterized by aggressive invasion and poor prognosis. Abnormal remodeling of the tumor microenvironment plays a pivotal role in its disease progression. The tumor stroma, which is a core component of the tumor microenvironment, comprises an extracellular matrix and various stromal cells. Changes in its composition are closely associated with tumor proliferation, invasion, metastasis, and drug resistance. Collagen Type VI alpha 1 chain (COL6A1), an important functional protein in the extracellular matrix, plays a carcinogenic role in multiple malignant tumors; it participates in matrix remodeling and cellular signaling and influences the tumor immune microenvironment. This systematic review aims to examine the biological properties of COL6A1 and the structural characteristics of CCA tumor stroma, and summarize the roles of stromal COL6A1 in CCA.

**Recent Findings:**

We outline the expression patterns of stromal COL6A1 in CCA and its correlation with clinicopathological features. Specifically, we systematically discuss the molecular mechanisms by which COL6A1 facilitates CCA progression, including mediating stromal remodeling, cancer‐associated fibroblast activation, oncogenic signaling cascades, the immunosuppressive microenvironment, and therapeutic resistance. COL6A1 shows translational potential as a diagnostic biomarker, staging indicator, and prognostic predictor for CCA.

**Conclusion:**

COL6A1 is a critical molecule regulating stromal remodeling and malignant progression of CCA. This review highlights the current research gaps and proposes prospective research directions, providing a theoretical foundation for future investigations and clinical translations using COL6A1 in CCA.

## 1 Introduction

The global incidence of cholangiocarcinoma (CCA), a malignant tumor of the biliary system, is increasing. It is classified into intrahepatic, perihilar, and distal CCA based on location. The latter two subtypes are collectively referred to as extrahepatic CCA, accounting for approximately 90% of all cases and predominantly affecting individuals aged 50–70 [1]. Males with CCA slightly outnumber females with CCA, and most patients are diagnosed at intermediate to advanced stages, with a poor prognosis and 5‐year survival rate of < 10% [2]. Surgical resection remains the primary treatment; however, most patients miss the opportunity for radical surgery owing to early‐stage insensitivity, aggressive tumor behavior, and high lymph node metastasis risk [3]. Additionally, the efficacy of chemoradiotherapy is limited. Therefore, elucidating the molecular mechanisms underlying CCA progression is crucial for developing novel diagnostic and therapeutic strategies [4].

The tumor microenvironment (TME) is a complex dynamic system that is essential for tumor cell survival. The tumor stroma, the structural foundation of this microenvironment, constitutes a substantial portion of the tumor tissue volume. It is pathologically characterized by severe fibrosis and enriched with extracellular matrix (ECM) and stromal cells, including cancer‐associated fibroblasts (CAFs) and immune cells. Dysregulation of stromal components is closely associated with the malignant phenotype of CCA [5]. These components interact with one another to establish a pro‐tumorigenic niche that supports tumor proliferation, invasion, and metastasis. The ECM is a central structural and signaling constituent of the stroma involved in tumor‐stroma crosstalk modulation [6, 7]. COL6A1, an important ECM protein, may play a key regulatory role in this process [8]; however, further research is required to elucidate its specific mechanisms.

The *COL6A1* gene is located on the human chromosome 21q22.3 and encodes the α1 chain of Type VI collagen. This protein forms heterotrimeric complexes with α2(VI) and α3(VI) chains, constituting the fundamental structural unit of Type VI collagen. It is widely distributed in the ECM of various tissues and plays a crucial role in maintaining tissue integrity, cell adhesion, and signal transduction [9]. COL6A1 is highly expressed in malignant tumors, such as osteosarcoma, pancreatic cancer, and sarcoma. Its expression levels are closely associated with tumor invasion, metastatic potential, and poor patient prognosis, primarily promoting tumor progression through mechanisms such as tumor stromal remodeling, oncogenic signaling pathway activation, and immune suppression [10]. These mechanistic insights are well supported in nonbiliary malignancies; however, experimental verification within the CCA is lacking. Compared to other malignancies, research on COL6A1 in CCA remains relatively scarce. Existing studies are primarily on the involvement of Type VI collagen in the CCA microenvironment. To the best of our knowledge, no systematic summary regarding its complete functional pathways and stromal interaction patterns is available [6, 11]. Therefore, systematically reviewing these research advances, exploring COL6A1 expression patterns, and hypothesizing the specific mechanisms in CCA are particularly important. In this review, the biological characteristics of COL6A1 and tumor stromal features of CCA are discussed. Specifically, the expression of COL6A1 in the tumor stroma and its hypothesized regulatory mechanisms in tumor progression are outlined. These findings provide new insights into molecular targeted therapies for CCA [12].

## 2 Composition and Biological Function of CCA Tumor Stroma

### 2.1 Clinical and Pathological Features and Progression of CCA

CCA, a malignant tumor originating from the epithelial cells of the intrahepatic and extrahepatic bile ducts, exhibits distinct geographical incidences. Its incidence is significantly higher in Asia than that in Europe and the United States. According to a National Cancer Center report, the annual incidence of extrahepatic CCA in China increased from 3.82 cases per 100 000 population in 2018 to 4.03 cases per 100 000 population in 2022, making it one of the most prevalent malignancies in the biliary system [13]. Most patients were aged 50–70, and males were slightly more affected than females. The exact etiology remains unclear but may involve risk factors, such as biliary stones, primary sclerosing cholangitis, liver fluke infection, and chronic ulcerative colitis. These factors induce chronic inflammation in the biliary epithelium and promote the malignant transformation of epithelial cells [14].

Pathologically, CCA predominantly manifests as adenocarcinoma, accounting for > 98% of cases. Well‐differentiated adenocarcinomas are the most common, whereas poorly differentiated and undifferentiated adenocarcinomas occur less frequently. Tumors can be classified into four main subtypes: papillary, nodular, sclerosing, and diffuse [15]. These subtypes exhibit distinct differences in clinical presentation, imaging features, surgical resection rates, and prognoses. A thorough understanding of these variations is essential to develop more precise and personalized treatment strategies [16]. Different tumor types exhibit distinct invasive and metastatic characteristics. Sclerosing CCA typically occurs in the hilar region of the liver, with substantial fibrosis. This tumor type is highly invasive and prone to invading neural and vascular tissues, making the condition more complex and difficult to control. In contrast, diffuse CCA primarily manifests as extensive thickening of the bile duct wall, leading to luminal narrowing and widespread infiltration into the surrounding tissues. Regarding the metastatic pathways, direct invasion and lymphatic metastasis are predominant, whereas hematogenous and implantation metastases are relatively rare. Tumors tend to infiltrate and spread along the tissues surrounding the bile ducts, nerves, lymphatic spaces, and blood vessels, thereby invading the liver parenchyma and adjacent organs. Moreover, the relatively high lymph node metastasis rate of CCA is a key factor contributing to the poor prognosis of patients [17].

The lack of specific early symptoms of CCA causes considerable challenges for its early diagnosis. Patients with intrahepatic CCA often experience nonspecific gastrointestinal symptoms, such as abdominal pain, bloating, and fatigue, which are easily overlooked or mistaken for common digestive disorders. In contrast, extrahepatic CCA typically manifests as progressive obstructive jaundice, characterized by dark urine, clay‐colored stools, and skin itching [18]. However, owing to the subtle early symptoms, most patients are diagnosed at intermediate or advanced stages, thereby missing the opportunity for curative surgery. The current treatment options for CCA remain limited. Although surgical resection is an option, the efficacy of radiotherapy and chemotherapy is limited. Targeted therapies, along with immunotherapy, are still in the experimental phase, resulting in low 5‐year survival rates. Therefore, in‐depth research into the molecular mechanisms driving CCA progression, particularly the regulatory role of the TME in tumor development, is crucial for improving patient outcomes.

### 2.2 Core Components of the CCA Tumor Stroma

The CCA tumor stroma constitutes an exceptionally complex heterogeneous system comprising the ECM and diverse stromal cells. These components interact dynamically and continuously remodel, collectively creating a tumor‐promoting microenvironment. Distinct from the normal biliary tissue stroma, the CCA stroma exhibits pronounced fibrosis, hypovascularization, and immunosuppression. Both the composition and proportion of its elements undergo considerable alterations, with the following core components being particularly noteworthy.

#### 2.2.1 Components of the ECM

The ECM, the primary structural component of the CCA tumor stroma, contains substances such as collagen, proteoglycans, and glycoproteins. Abnormal collagen deposition is the main cause of stromal fibrosis in tumors. The CCA stroma is rich in collagen Types I, III, and VI. Their excessive synthesis and deposition increase stromal stiffness, providing mechanical support for tumor cell invasion and regulating tumor cell behavior through signal transduction [19]. In addition to collagen, the stroma contains glycoproteins, such as fibronectin, laminin, perivin, and tendinogen‐C, as well as proteoglycans, such as hyaluronic acid and chondroitin sulfate. These components are involved in stromal remodeling and signal transduction by binding to or interacting with cell‐surface receptors, thereby promoting tumor progression [20].

Furthermore, the CCA tumor stroma contains multiple proteases capable of regulating ECM remodeling, including matrix metalloproteinases (MMPs) and their tissue inhibitors. Notably, MMP‐9 is highly expressed in CCA and closely associated with tumor development, disease progression, and prognosis. By degrading ECM components and disrupting tissue barriers, MMP‐9 facilitates tumor cell invasion and metastasis. It also plays a critical role in angiogenesis and immune cell infiltration during tumor progression. Hypoxia, a key microenvironmental factor and defining characteristic of CCA stroma, further promotes tumor and stromal cell proliferation as well as angiogenesis through the activation of key regulatory molecules, such as hypoxia‐inducible factor (HIF) [21].

#### 2.2.2 Components of the Stromal Cells

The tumor stroma in biliary tract cancer contains an exceptionally diverse array of stromal cells, including CAFs, immune cells, hepatic stellate cells (HSCs), and mesenchymal stem cells (MSCs). These stromal cell types play a pivotal role in maintaining homeostasis of the TME and promoting tumor progression by secreting various cytokines, chemokines, and ECM components. Among these, CAFs are one of the most dominant stromal cell populations originating from resident fibroblasts, HSCs, and MSCs. These cells are activated by cytokines, such as transforming growth factor‐β (TGF‐β) and interleukin‐6 (IL‐6) secreted by tumor cells, and express signature proteins, including α‐smooth muscle actin (α‐SMA), fibroblast‐activated protein (FAP), and vimentin [22, 23].

Immune cells, including tumor‐associated macrophages (TAMs), regulatory T cells (Tregs), myeloid‐derived suppressor cells (MDSCs), and neutrophils, are crucial components of the CCA tumor stroma. These immune cells predominantly exhibit immunosuppressive phenotypes that collectively create an immunosuppressive TME. In CCA, M2‐type TAMs extensively infiltrate the TME [24]. Additionally, MDSCs inhibit cytotoxic T cells and natural killer (NK) cells by expressing arginase‐1 and nitric oxide synthase‐2 [25]. Furthermore, HSCs activate the Hh signaling pathway through ECM components [26].

### 2.3 The Biological Functions of the CCA Tumor Stromata and Their Impact on Tumor Progression

The tumor stroma of biliary tract carcinoma is not only a passive structural support system but actively regulates tumor progression through multiple complex mechanisms. Its biological functions primarily involve promoting the formation of malignant tumor cell phenotypes, modulating the immune microenvironment, and enhancing treatment resistance. Its specific roles are outlined in the following sections.

#### 2.3.1 Promoting Tumor Cell Proliferation, Invasion, and Metastasis

The tumor stroma considerably enhances the proliferative activity and invasive metastatic capacity of CCA cells by providing essential mechanical support and precise signal regulation. Excessive deposition of components, such as collagen, in the stroma increases stromal stiffness. Enhanced stiffness can induce cytoskeletal reorganization in tumor cells via mechanical signaling, thereby promoting cellular migration. Simultaneously, this increased stiffness establishes physical pathways for tumor cell invasion, facilitating the breakthrough of the original tissue barriers. Stromal cells, such as CAFs, secrete pro‐oncogenic factors, including TGF‐β, hepatocyte growth factor, and epidermal growth factor. When these factors bind to receptors and specific ligands on tumor cell surfaces, they activate downstream signaling pathways, such as PI3K/AKT and MAPK. These activated signaling pathways play crucial roles in promoting tumor cell proliferation. Additionally, this signaling cascade can induce epithelial–mesenchymal transition (EMT). EMT initiation leads to significant morphological and functional changes in tumor cells, markedly enhancing their invasive and metastatic capabilities [27]. Furthermore, proteases, such as MMPs, degrade the ECM. This degradation breaks down the original tissue barrier structure, creating favorable conditions for tumor cells to penetrate the basement membrane, infiltrate surrounding tissues, and metastasize to distant sites [28].

#### 2.3.2 Constructing an Immunosuppressive Microenvironment to Promote Tumor Immune Evasion

The tumor stroma in biliary tract cancer regulates immune cell infiltration and function through multiple mechanisms, creating a unique immunosuppressive microenvironment. This environment effectively enables tumor cells to evade antitumor immune surveillance. Within the tumor stroma, CAFs secrete immunosuppressive factors, such as IL‐6 and IL‐10. These factors recruit Tregs and MDSCs, facilitating their infiltration into tumor tissues. Simultaneously, they inhibit the activation and proliferation of antitumor immune cells, such as effector T and NK cells. TAMs infiltrating tumor tissues suppress immune cell activity by secreting pro‐inflammatory cytokines and expressing immune checkpoint molecules, thereby weakening the body's immune response [29]. Additionally, abnormal deposition of ECM components in the tumor stroma hinders immune cell infiltration into tumor cells, reducing immunotherapy efficacy and collectively promoting tumor immune evasion and progression.

#### 2.3.3 Enhancing Tumor Resistance to Treatment

The tumor stroma in biliary tract cancer is a key factor contributing to the resistance to radiotherapy, chemotherapy, and targeted therapies. The highly fibrotic stroma is a robust physical barrier that hinders the delivery of chemotherapeutic agents and targeted drugs into tumor cells, resulting in reduced drug concentrations within tumor cells and diminished treatment efficacy. In contrast, stromal cells regulate the drug sensitivity of tumor cells by secreting various cytokines. For instance, TGF‐β secreted by CAFs activates drug‐resistant signaling pathways in tumor cells, upregulates expression levels of multidrug‐resistant genes, and thereby enhances cellular resistance to chemotherapy drugs. Additionally, the hypoxic environment in the tumor stroma induces the expression of molecules, such as HIF‐1α [10]. The expression of these molecules enables tumor cells to adapt to stressful conditions and increases their tolerance to radiotherapy and chemotherapy, ultimately leading to reduced treatment efficacy.

## 3 Biological Characteristics of COL6A1

### 3.1 COL6A1 Gene and Protein Structure

*COL6A1* is a key gene in the Type VI collagen family, located in a specific region of human chromosome 21q22.3. The translated product of this gene forms the α1 chain of Type VI collagen. This polypeptide chain contains 1028 amino acid residues, has a relative molecular mass of approximately 108.5 kDa, and exhibits typical collagen structural characteristics. The fundamental functional unit of Type VI collagen is a heterotrimer, which consists of the α1 chain encoded by COL6A1, the α2 chain encoded by COL6A2, and the α3 chain encoded by COL6A3, covalently linked together. These trimers further assemble into dimers and tetramers, ultimately forming a characteristic bead‐like network structure in the ECM. This structure is widely distributed in the basement membranes and interstitial spaces of various tissues, such as the skin, muscles, liver, and pancreas, and is a crucial structural foundation for maintaining tissue mechanical stability [30].

The α1 chain contains both collagenous and non‐collagenous domains. The collagenous domain, which is rich in glycine, is essential for forming the unique triple‐helical structure of collagen and plays an important role in maintaining its stability and functionality [31]. The glycine–proline–hydroxyproline repeat units in the amino acid sequence form the core region of the triple‐helical structure. In contrast, the non‐collagenous domain is crucial for protein–protein interactions and signal molecule binding, providing a structural foundation for cell adhesion and signal transduction. Additionally, the non‐collagenous domain on the α1 chain can specifically bind to ECM components (such as laminin and fibronectin) and cell surface receptors (such as integrin family proteins), thereby constructing complex matrix networks that transmit extracellular signals and regulate various biological behaviors, including cell proliferation, differentiation, and migration [32].

### 3.2 Physiological Functions of COL6A1

Under normal physiological conditions, Type VI collagen is a vital functional component of the ECM. It plays crucial roles in maintaining tissue integrity, regulating cellular behavior, and facilitating tissue repair. Collagen fibers interwoven into a reticular structure significantly enhance the mechanical strength and elasticity of the ECM. This characteristic is particularly prominent in high‐load‐bearing tissues, such as muscles and tendons, where abundant Type VI collagen is essential for maintaining tissue stability. Mutations in COL6A1 disrupt Type VI collagen synthesis, leading to muscular connective tissue disorders, such as Bethlem myopathy and congenital Ullrich‐type muscular dystrophy. This phenomenon further underscores the central role of proteins in maintaining tissue homeostasis [33].

Cell–matrix interactions mediated by COL6A1 constitute a pivotal mechanism for regulating cellular physiological functions. Type VI collagen binds to cell surface integrin receptors (α1β1 and α2β1) through its α1 chain non‐collagenic domain, thereby activating downstream signaling pathways to facilitate cell adhesion and spreading and maintain normal cellular morphology and polarity [34]. During tissue injury repair, COL6A1 expression significantly increases, thereby accelerating tissue regeneration by promoting fibroblast proliferation, matrix component synthesis, and angiogenesis. However, abnormal expression may lead to excessive repair, potentially causing pathological conditions, such as tissue fibrosis.

Furthermore, COL6A1 is involved in the regulation of multiple physiological signaling pathways, including the classical Wnt, apoptosis, and autophagy pathways. It maintains the cellular metabolic balance and tissue homeostasis by modulating the activity of these pathways. For instance, COL6A1 regulates the Wnt signaling pathway and participates in bone development and cartilage formation. Abnormal expression of COL6A1 disrupts the normal differentiation and maturation of bone tissue [35]. Additionally, the signaling mediated by COL6A1 regulates programmed cell death, which is crucial for maintaining a normal balance between cell survival and death.

### 3.3 Abnormal Expression and Function of COL6A1 in Diseases

Abnormal COL6A1 expression is closely associated with various human diseases, particularly in pathological processes such as tumors, fibrotic disorders, and skeletal diseases, where its expression levels are significantly correlated with clinical pathological features and prognosis. In fibrotic diseases, such as pulmonary and hepatic fibrosis, COL6A1, a key matrix synthesis‐related gene, is highly expressed in tissues. For instance, the highly expressed FAP in pulmonary fibrotic tissues shares similar mechanisms with those of COL6A1, both accelerating fibrosis by promoting collagen deposition and matrix remodeling [36]. ADAMTS‐1 levels change in the serum of rats with experimental pulmonary fibrosis, demonstrating its potential influence on the progression of pulmonary fibrosis. This phenomenon is consistent with the mechanism of action of COL6A1 in pulmonary fibrotic tissues. Specifically, COL6A1 can induce fibroblast activation and increase the synthesis of ECM components, ultimately leading to more severe structural damage in lung tissue [37].

In oncology research, the abnormal overexpression of COL6A1 was observed in various malignant tumors, with its expression levels showing a highly significant correlation with tumor malignancy. *COL6A1* gene expression is markedly elevated in osteosarcoma tissues, particularly at specific sites, such as lung metastases [38]. The strong association between COL6A1 overexpression and poor overall survival may stem from two mechanisms: inhibition of the STAT1 signaling pathway and activation of CAFs in lung tissues, thereby facilitating tumor metastasis. In pancreatic cancer tissues, COL6A1 expression levels were > 10 times higher than those in adjacent normal tissues. This hyper‐expression is closely linked to severe symptoms, including distant metastasis and vascular invasion, and an independent prognostic risk factor for patient survival. Silencing COL6A1 expression can significantly suppress the migratory and invasive capabilities of pancreatic cancer cells and reverse the progression of EMT [39]. In sarcoma tissues, whether osteosarcoma or soft tissue sarcoma, COL6A1 consistently shows high expression levels that are positively correlated with neutrophil infiltration. Patients with COL6A1 overexpression demonstrated significantly reduced overall survival and disease‐specific survival rates, establishing COL6A1 as a potential prognostic biomarker and therapeutic target for sarcoma management [40].

Furthermore, polymorphic sites of the *COL6A1* gene are associated with an increased risk of yellow ligament ossification and posterior longitudinal ligament ossification (OPLL), making it a susceptibility gene for these conditions in the Han population. BMP2, BMP4, BMP7, TGF‐β, and OPLL are correlated, with the *COL6A1* gene playing a particularly crucial role in the pathogenesis of OPLL. In patients with ankylosing spondylitis, genetic variations in COL6A1 may be linked to uveitis development, suggesting that this gene regulates the progression of autoimmune diseases [41]. These findings conclusively demonstrate that abnormal COL6A1 expression occurs in the pathogenesis of multiple diseases, with its mechanisms exhibiting distinct tissue‐specific characteristics. Targeting COL6A1 may be a novel therapeutic strategy for related disorders.

## 4 COL6A1 Expression in the CCA Tumor Matrix

### 4.1 COL6A1 Expression in the Tumor Tissue and Stroma

To date, studies on the expression of COL6A1 in CCA remain relatively limited. In existing research, COL6A1 exhibits significantly high expression in malignant digestive system tumors, such as pancreatic and liver cancers, is primarily localized in the tumor stroma, and its expression levels positively correlate with the degree of stromal fibrosis [38, 39]. As an important type of digestive system tumor, CCA has a substantially fibrotic stroma rich in ECM components, such as collagen. Therefore, the expression level of COL6A1 in CCA tissue may be considerably higher than that in normal bile duct and adjacent normal tissues and predominantly distributed in the tumor stroma.

In studies on pancreatic cancer and osteosarcoma, COL6A1 is primarily secreted by activated CAFs, with low expression levels in tumor cells [42]. Silencing COL6A1 in CAFs significantly inhibits tumor progression. In CCA, CAFs, the primary ECM‐producing cells in the stroma, become highly activated under cytokine induction, such as TGF‐β. These cells are hypothesized to be the main source of COL6A1, which contributes to tumor stromal remodeling through collagen secretion. Additionally, HSC‐specific stromal cells in the CCA can synthesize large amounts of collagen and other ECM components upon activation. Their involvement in COL6A1 expression and secretion likely enhances its accumulation within the tumor stroma [43, 44, 45].

### 4.2 COL6A1 Expression and Clinical Pathological Parameters of CCA

Based on previous studies on COL6A1 in other cancers, we hypothesized that its expression level in CCA is closely associated with clinicopathological parameters and may serve as a potential biomarker for assessing tumor malignancy.

In pancreatic cancer, high COL6A1 expression is significantly linked to distant metastasis and vascular invasion and is an independent prognostic risk factor for overall patient survival. Similarly, elevated COL6A1 expression is associated with pulmonary metastasis and low overall and disease‐specific survival for sarcoma, particularly osteosarcoma [37, 39, 46]. Therefore, high COL6A1 expression in CCA may be associated with malignant pathological features, such as tumor stage, differentiation degree, lymph node metastasis, and vascular invasion. In tumor staging, patients with advanced CCA (Stages III and IV) exhibit more severe stromal fibrosis, with potentially significantly higher COL6A1 expression levels compared with those in early‐stage patients (Stages I and II) [10, 47]. Poorly differentiated CCA demonstrates greater malignancy and more pronounced stromal remodeling than those of well‐ or moderately differentiated variants, often accompanied by elevated COL6A1 expression [48]. Regarding metastatic characteristics, patients with lymph node or distant metastases showed markedly increased COL6A1 expression in the tumor stroma, which positively correlates with tumor invasion and metastasis potential. Additionally, COL6A1 expression may correlate with pathological parameters, such as tumor size, vascular invasion, and neural invasion. Patients with high COL6A1 expression typically exhibit stronger tumor invasiveness and higher malignancy [49].

A clear link between COL6A1 and CCA prognosis has not been established; however, research on the expression patterns of related genes, such as *CCDC6* and *SPARCL1*, along with their prognostic value, provides insights into the potential role of COL6A1 in predicting disease outcomes. Future large‐scale clinical trials will involve immunohistochemical and qRT‐PCR methods to analyze COL6A1 expression in CCA tissues and confirm its significance in disease progression and prognostic evaluation.

### 4.3 COL6A1 Expression in Different CCA Subtypes and Models

Different CCA subtypes exhibit significant variations in their pathological locations, biological characteristics, and prognostic outcomes, with distinct tumor stromal compositions. Therefore, COL6A1 expression may vary across subtypes. Specifically, intrahepatic CCA originates from epithelial cells in secondary branches or distal intrahepatic bile ducts, showing more pronounced activation of HSCs in the stroma. In contrast, hilar CCA predominantly exhibits sclerosing features with high stromal fibrosis and abundant CAFs. Distal CCA is mainly papillary or nodular with relatively sparse stromal components [50]. Therefore, COL6A1 expression may be the highest in hilar CCA, followed by intrahepatic CCA, and the lowest expression in distal CCA. This variation likely closely correlates with the degree of stromal fibrosis and CAF infiltration in each subtype.

Differences in COL6A1 expression may exist between the cellular and animal models. For instance, in a coculture model of CAFs using highly invasive CCA (e.g., QBC939 and FRH‐0201) and less invasive (e.g., HCCC‐9810) cell lines, highly invasive tumor cells may more readily activate CAFs, leading to elevated COL6A1 expression. In CCA–transplanted tumor models in nude mice, metastatic tumor tissues may demonstrate significantly higher COL6A1 expression than non‐metastatic models, with more concentrated expression in stromal regions [51]. Additionally, in animal models of CCA liver metastasis, metastatic lesions may exhibit higher COL6A1 expression than primary tumors, which is correlated with tumor metastasis progression. These findings align with the high COL6A1 expression observed in lung metastases of osteosarcoma and correspond to the differential gene expression profiles of primary liver cancer and CCA thrombus samples.

## 5 Molecular Mechanisms of COL6A1 in the Progression of CCA

COL6A1 drives malignant progression of CCA via multiple synergistic pathways. The comprehensive regulatory network is shown in Figure 1.

**FIGURE 1:**
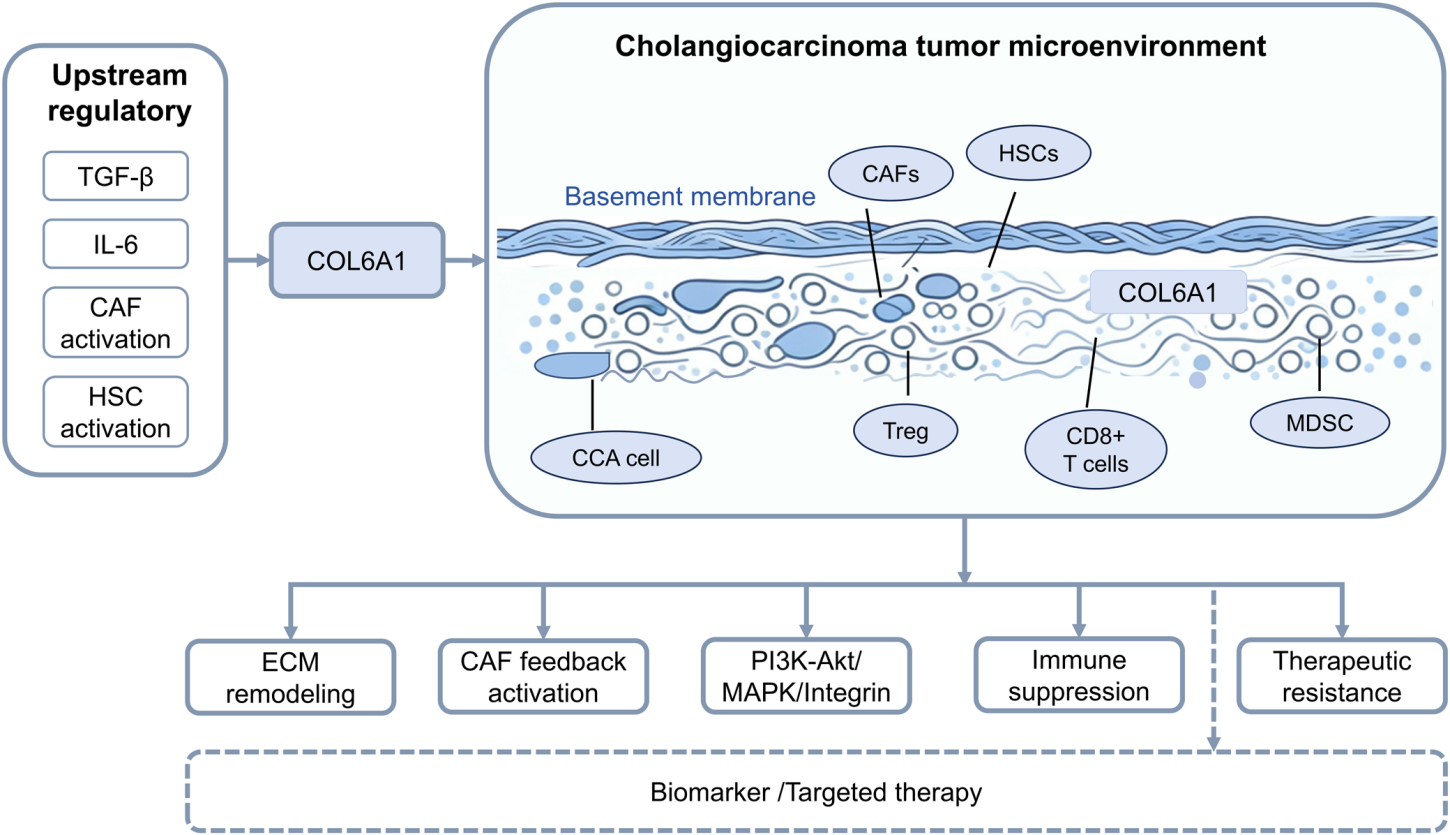
Schematic diagram of the COL6A1 regulatory network within the cholangiocarcinoma (CCA) microenvironment. Upstream stimuli, including TGF‐β, IL‐6, as well as activated CAFs and HSCs, induce the expression and secretion of COL6A1. Secreted COL6A1 is released into the tumor stroma of cholangiocarcinoma, driving ECM remodeling and activating the PI3K‐Akt/MAPK signaling via integrins. It mediates positive‐feedback activation of CAFs, facilitates the recruitment of MDSCs and Tregs, and triggers CD8^+^ T‐cell‐mediated immune suppression, which ultimately contributes to therapeutic resistance. COL6A1 may serve as a potential biomarker and therapeutic target for cholangiocarcinoma. Thin black lead lines denote different cell types. Partial mechanistic evidence is derived from other tumor types, which remains to be further validated by in vitro and in vivo experiments in cholangiocarcinoma.

### 5.1 Mediating Tumor Matrix Remodeling and Enhancing Mechanical Support

As a crucial component of the ECM, COL6A1 plays a role in the synthesis and remodeling of the tumor stroma in CCA by altering its physical properties, thereby supporting tumor progression. Secreted by stromal cells, such as CAFs, COL6A1 assembles with α2(VI) and α3(VI) chains to form Type VI collagen. Collagen then interweaves with other ECM components, including Types I and III collagen and fibronectin, creating a dense network that promotes stromal deposition and intensifies tumor stromal fibrosis. The resulting stromal fibrosis significantly increases matrix stiffness, which influences tumor cell behavior via mechanical signaling pathways. These pathways induce cytoskeletal reorganization and enhance cellular morphological changes and migration capacity, and simultaneously boost the invasive ability of tumor cells into surrounding tissues [52].

Furthermore, COL6A1 regulates the matrix structure by modulating the activity of proteases involved in ECM remodeling. Col6a1 is closely associated with MMP activity in pancreatic cancer; the knockdown of Col6a1 can lead to downregulated MMP2 and MMP9 expression and inhibited ECM degradation and tumor invasion [53]. Accordingly, COL6A1 expression may indicate that MMP expression and activity locally degrade the ECM architecture and disrupt basement membrane barriers in CCA, establishing favorable conditions for tumor cell invasion and metastasis. Additionally, COL6A1 may bind to laminin to further impair the structural integrity of the basement membrane and accelerate tumor progression.

### 5.2 Regulation of CAF Activation and Function

CAFs, the predominant stromal cell type in biliary CCA, exhibit activation states that are correlated with tumor progression. COL6A1 regulates CAF activation, proliferation, and secretory functions, thereby indirectly driving CCA progression. COL6A1 drives the phenotypic transformation of quiescent fibroblasts into CAFs and upregulates the expression of canonical CAF markers, including α‐SMA and FAP. COL6A1 promotes CAF proliferation to expand the pool of activated stromal fibroblasts [54]. Additionally, COL6A1 remodels the secretory profile of CAFs, triggering the robust secretion of oncogenic mediators, including TGF‐β, CTGF, and SDF‐1, as well as several ECM components. These factors act on CCA cells in a paracrine manner to aggravate malignant phenotypes and exacerbate stromal fibrosis. Moreover, activated CAFs secrete MMPs to drive stromal remodeling, and COL6A1 upregulates intracellular MMP2 and MMP9 expression to promote ECM degradation [55]. Collectively, COL6A1 may facilitate CCA progression by driving the transformation of resident fibroblasts into pro‐tumor CAFs, reprogramming the pro‐oncogenic secretory phenotype of CAFs to construct a COL6A1–CAF–tumor cell positive feedback loop, and synergistically increasing MMP levels to remodel the ECM.

### 5.3 Activating Downstream Signaling Pathways to Regulate Tumor Cell Biology

COL6A1 activates downstream oncogenic signaling pathways by binding to receptors on tumor cell surfaces, directly regulating biological behaviors, such as proliferation, apoptosis, invasion, and metastasis in CCA cells. The Type VI collagen complex formed by COL6A1 can interact with tumor cells expressing multiple integrin receptors, including α1β1 and α2β1, that specifically bind to ligands [56]. COL6A1 regulates tumor proliferation, apoptosis, invasion, and metastasis mainly through the PI3K/AKT and MAPK pathways. In sarcoma, COL6A1 co‐expressed genes are significantly enriched in the PI3K/AKT and MAPK signaling axes [57]. The activated PI3K/AKT/mTOR pathway facilitates cell cycle progression, inhibits apoptosis, and promotes tumor invasion and metastasis. In pancreatic cancer, COL6A1 positively regulates EMT, and COL6A1 silencing upregulates E‐cadherin and downregulates vimentin, thereby reversing the EMT process [58]. COL6A1 inhibits the PI3K/AKT signaling pathway in bladder cancer, thereby suppressing tumor cell proliferation and migration [59]. However, this effect may be tumor‐specific, as COL6A1 tends to activate cancer‐promoting pathways in most tumor types. Furthermore, in osteosarcoma, COL6A1 interacts with SOCS5, acting as a catalyst to promote the ubiquitination and degradation of STAT1, thereby inhibiting the STAT1 signaling pathway and facilitating tumor metastasis [60].

Therefore, COL6A1 may activate PI3K/AKT and MAPK signaling via integrin binding to promote proliferation, inhibit apoptosis, induce EMT, and enhance the invasive and metastatic capacities of CCA cells [61]. Additionally, it may modulate the STAT1 pathway to affect tumor progression. However, these potential mechanisms remain to be experimentally validated in patients with CCA.

### 5.4 Modulating the Tumor Immune Microenvironment and Promoting Immune Escape

COL6A1 facilitates immune evasion by creating an immunosuppressive microenvironment through the regulation of immune cell infiltration and functional status of the CCA tumor stroma. COL6A1 expression is significantly increased in sarcoma tissues, with a strong positive correlation with neutrophil infiltration (*r* = 0.280; *p* < 0.05). The co‐expression of COL6A1 with genes involved in immune cell infiltration suggests its potential role in modulating tumor immunity via immune cell regulation [62, 63].

The immunomodulatory effects of COL6A1 were previously confirmed. First, COL6A1 recruits and activates immunosuppressive cells, including MDSCs and Tregs. MDSCs suppress the activity of effector T cells by secreting arginase‐1 and NOS2 to deplete essential nutrients and produce inhibitory metabolites. Tregs secrete anti‐inflammatory cytokines, IL‐10 and TGF‐β, to dampen antitumor immune responses. COL6A1 promotes the accumulation of immunosuppressive cells by regulating chemokine signaling axes [64]. Second, COL6A1 inhibits the proliferation and activation of effector T and NK cells, impairing their recognition and cytotoxicity against tumor cells. It also drives the polarization of TAMs toward the pro‐tumor M2 phenotype to support tumor growth, angiogenesis, and immune evasion. Furthermore, COL6A1‐induced stromal fibrosis forms a dense physical barrier that blocks the infiltration of functional immune cells into the tumor parenchyma and weakens systemic antitumor immunity, creating favorable conditions for tumor immune evasion and progression.

COL6A1 may facilitate immune escape by recruiting and activating MDSCs and Tregs, inhibiting effector lymphocyte function, inducing M2 polarization of TAMs, and establishing physical barriers through stromal fibrosis. Further in vitro and in vivo experiments are required to verify the regulatory mechanism of CCA.

### 5.5 Development of Drug Resistance in CCA

COL6A1 may significantly contribute to the development of drug resistance in CCA by modulating tumor stromal characteristics and altering intracellular signaling pathways, thereby substantially enhancing the tumor resistance to therapeutic interventions. The highly fibrotic nature of CCA stroma is a key factor in treatment resistance, and COL6A1 is a critical regulator of stromal fibrosis.

Specifically, COL6A1 promotes the deposition of ECM components, forming a dense and impermeable physical barrier. This not only impedes the effective delivery of chemotherapeutic and targeted drugs into tumor cells, but also reduces their concentration distribution within tumor tissues, thereby significantly diminishing treatment efficacy [65]. CAF‐derived TGF‐β activates SMAD‐dependent signaling and upregulates MDR1 expression, which enhances chemoresistance in pancreatic ductal adenocarcinoma [66, 67, 68]. Additionally, the COL6A1‐activated PI3K/AKT pathway upregulates anti‐apoptotic Bcl‐2 family proteins and desensitizes tumor cells to drug‐induced apoptosis [69, 70]. COL6A1‐related microenvironmental disorders also activate HIF‐1α signaling, leading to metabolic reprogramming and increased radioresistance and chemoresistance [71, 72].

Given the highly fibrotic and hypoxic microenvironment of CCA, COL6A1 may synergistically trigger drug resistance by promoting ECM accumulation to form a physical drug barrier. This activates the CAF–TGF‐β axis to upregulate drug‐resistant genes and stimulates the PI3K/AKT/HIF‐1α pathway to inhibit drug‐induced apoptosis and remodel tumor metabolism, thereby attenuating the responsiveness of CCA to radiotherapy, chemotherapy, and targeted therapy.

## 6 Clinical Application Potential of COL6A1 for CCA

COL6A1 drives multiple oncogenic cascades, including stromal fibrosis, CAF activation, immunosuppression, and chemoresistance (Figure 1). Therefore, it is a potential target for the diagnosis and treatment of CCA.

### 6.1 Potential Biomarkers for CCA Diagnosis and Staging

Despite the current challenges in the early diagnosis of CCA, the lack of specific biomarkers remains a critical issue. Although clinically used markers, such as CA19‐9 and CEA, demonstrate diagnostic value, their limitations in sensitivity and specificity fail to meet early detection requirements [73]. Therefore, novel approaches, including microRNA and long noncoding RNA biomarkers, are being studied for enhanced diagnostic accuracy. Advanced techniques, such as multimodal and molecular imaging, are also being integrated into early diagnostic strategies. As a tumor stromal‐associated gene, *COL6A1* is highly expressed in CCA tissues, and its expression levels are closely correlated with tumor staging. In other cancers, such as pancreatic carcinoma and osteosarcoma, COL6A1 expression can be detected through tissue biopsies or blood tests, and its levels directly reflect tumor malignancy and aid in diagnostic and staging assessments [74, 75]. However, there are currently no large‐scale clinical cohort studies to confirm the diagnostic efficacy of COL6A1 in CCA. Currently, direct clinical diagnosis, screening of high‐risk populations, and staging evaluation are not supported; they only have potential research value at the basic research level.

COL6A1 may be used for diagnosing CCA using three approaches. The first strategy involves standardized molecular methods, including immunohistochemistry and qRT‐PCR, to quantify COL6A1 expression levels in tumor tissues. These results can be combined with imaging findings to support tumor confirmation and pathological staging. However, a unified interpretation standard and control baseline for benign biliary diseases have not been established. The second strategy involves developing blood‐based assays to measure serum COL6A1 protein or mRNA levels, which are noninvasive diagnostic markers for high‐risk population screening and early detection. This method is convenient, with minimal physical strain on patients; however, there is still no standardized test kit or reference threshold for healthy individuals. The third strategy involves combining COL6A1 with traditional tumor markers (e.g., CA19‐9) to enhance the diagnostic sensitivity and specificity. To translate COL6A1 into clinical practice, a prospective large‐sample multicenter clinical study must be conducted to systematically verify its diagnostic sensitivity, specificity, and differential capacity for benign and malignant biliary lesions. Sufficient clinical evidence is a prerequisite for defining optimal diagnostic cutoffs, establishing reliable combinatorial detection algorithms, and ultimately supporting clinical utility. Extensive research is required to validate the practical value of COL6A1in CCA diagnosis and staging and develop combination testing protocols for clinical application.

### 6.2 Indicator for CCA Prognosis Assessment

COL6A1 is a prognostic marker in multiple tumor types and an independent risk factor [76]. Elevated COL6A1 expression in pancreatic cancer directly correlates with significantly reduced median survival, with the protein as an independent predictor of overall survival. In osteosarcoma, high COL6A1 levels are strongly associated with pulmonary metastasis and poor prognosis, suggesting its crucial role in tumor invasion and metastasis. Similarly, increased COL6A1 expression in sarcoma tissues correlates with poorer overall and disease‐specific survival rates, indicating its prognostic value [77, 78]. Therefore, it may also have important prognostic value in CCA. If future studies confirm that patients with high COL6A1 expression in CCA have worse outcomes, this biomarker could become a valuable prognostic indicator for risk stratification and treatment optimization, potentially improving the survival rates and quality of life of affected patients.

In theory, clinicians could develop more accurate prognostic models that detect the expression levels of COL6A1 and other biomarkers (such as CEACAM6, SPARCL1, CXCL6, and IL‐6) in tumor tissues or serum samples from patients with CCA, combined with detailed clinicopathological parameters. This approach may enable physicians to better assess patient outcomes and provide robust support for personalized treatment strategies. It may help formulate optimal treatment plans tailored to individual patients, thereby enhancing therapeutic efficacy and precision. However, this model remains theoretical without validation for clinical specimens. If subsequent studies verify that COL6A1 can stratify patient prognosis, high‐risk patients with elevated COL6A1 expression may require increased postoperative follow‐up frequency or intensified adjuvant therapy to improve prognosis. Conversely, low‐risk patients with low COL6A1 expression can adopt standard treatment protocols and follow‐up strategies, effectively avoiding overtreatment and minimizing the need for limited medical resources [79]. Additionally, COL6A1 has potential as a biomarker for evaluating treatment response. The post‐treatment detection of reduced COL6A1 expression may indicate a better therapeutic response and more favorable prognosis. All these assumptions require validation using clinical data.

### 6.3 Potential Therapeutic Target for CCA

COL6A1 plays a pivotal role in promoting tumor progression. It participates in the regulation of stromal remodeling, activation of CAFs, and downstream signaling cascades, thus conferring its theoretical potential as a therapeutic target [80]. Current CCA therapeutic approaches primarily involve three aspects: inhibiting gene expression, blocking receptor binding, and suppressing downstream signaling pathways. These strategies have demonstrated therapeutic potential against other tumor types. For instance, silencing COL6A1 significantly reduces osteosarcoma tumor cell migration, invasion capacity, and lung metastasis. Its silencing diminishes pancreatic cancer tumor cell invasiveness, thereby improving patient prognosis. However, the therapeutic targeting of COL6A1 in CCA is still only a scientific hypothesis that has not been translated into clinical practice. If this strategy is verified in clinical trials, COL6A1‐targeted therapy may be valuable for CCA treatment.

Therapeutic strategies targeting COL6A1 could be further explored for CCA. First, advanced techniques, such as RNA interference and gene editing, may be used to silence COL6A1 expression, thereby reducing protein synthesis. This mechanism may inhibit tumor stromal remodeling and CAF activation, ultimately curbing tumor progression. Second, specific antibodies or inhibitors targeting the COL6A1 protein are required. These agents block the binding of COL6A1 to cell surface receptors, thereby suppressing downstream oncogenic signaling pathways and mitigating malignant phenotypes in tumor cells. Third, integrating COL6A1‐targeted therapies with existing treatments, such as chemotherapy, targeted therapy, and immunotherapy, can enhance the overall efficacy. By improving the tumor stromal microenvironment through COL6A1 inhibition, this approach not only boosts the effectiveness of current therapies, but also helps reverse tumor cell resistance. For instance, FGFR inhibitors, such as pemetrexed and infigratinib, have demonstrated promising clinical outcomes in patients with CCA. Combining COL6A1 inhibitors with chemotherapy drugs can enhance drug delivery efficiency by disrupting the physical barrier of the tumor stroma and inhibiting drug‐resistant signaling pathways to improve treatment outcomes. When used in combination with immunotherapies, these inhibitors can reshape the tumor immune microenvironment, boost the antitumor immune response, and ultimately enhance the overall efficacy of immunotherapy [81, 82]. However, these therapeutic strategies remain theoretical, lack systematic experimental verification in CCA models, and have not been translated into clinical practice.

## 7 Research Prospects and Limitations

### 7.1 Limitations of Existing Research

Although the role of COL6A1 has been partially investigated in various cancers, its role in CCA remains underexplored, severely limiting its clinical application. Notably, direct research targeting COL6A1 in CCA remains scarce, and no systematic studies on its expression patterns or functional mechanisms exist. Most available evidence is from scattered high‐throughput screening data without targeted validation, and the majority of the regulatory pathways outlined have been extrapolated from strategies for other malignancies. Additionally, the molecular mechanisms of COL6A1 in regulating biliary CCA progression remain unclear. Key downstream signaling pathways and detailed interactions with stromal and immune cells are yet to be elucidated, particularly regarding their role in immune microenvironment regulation and drug resistance development, for which there is a lack of experimental evidence. Third, clinical translational research on biliary CCA remains underdeveloped. Currently, clinical cohorts to evaluate the feasibility of Col6a1 as a diagnostic marker, prognostic indicator, or therapeutic target for CCA are limited. Furthermore, it is unclear whether it can be used in clinical staging and prediction of curative effects. Moreover, physiologically relevant research models are limited. Conventional monolayer cell models fail to recapitulate the highly fibrotic stromal microenvironment of CCA and cannot reproduce COL6A1‐mediated ECM interactions, restricting the accurate exploration of its in vivo functions. Furthermore, multilayer validation studies with public transcriptomic and spatial omics datasets are limited, and spatial information regarding COL6A1 localization and cell–cell crosstalk remains unavailable.

### 7.2 Future Research Directions

To address existing research limitations, a series of follow‐up investigations are required. Multiple in vitro, in vivo, and high‐throughput sequencing platforms are available to verify the regulatory mechanisms of COL6A1. Figure 2 shows an overview of the corresponding research models and analytical techniques.

**FIGURE 2:**
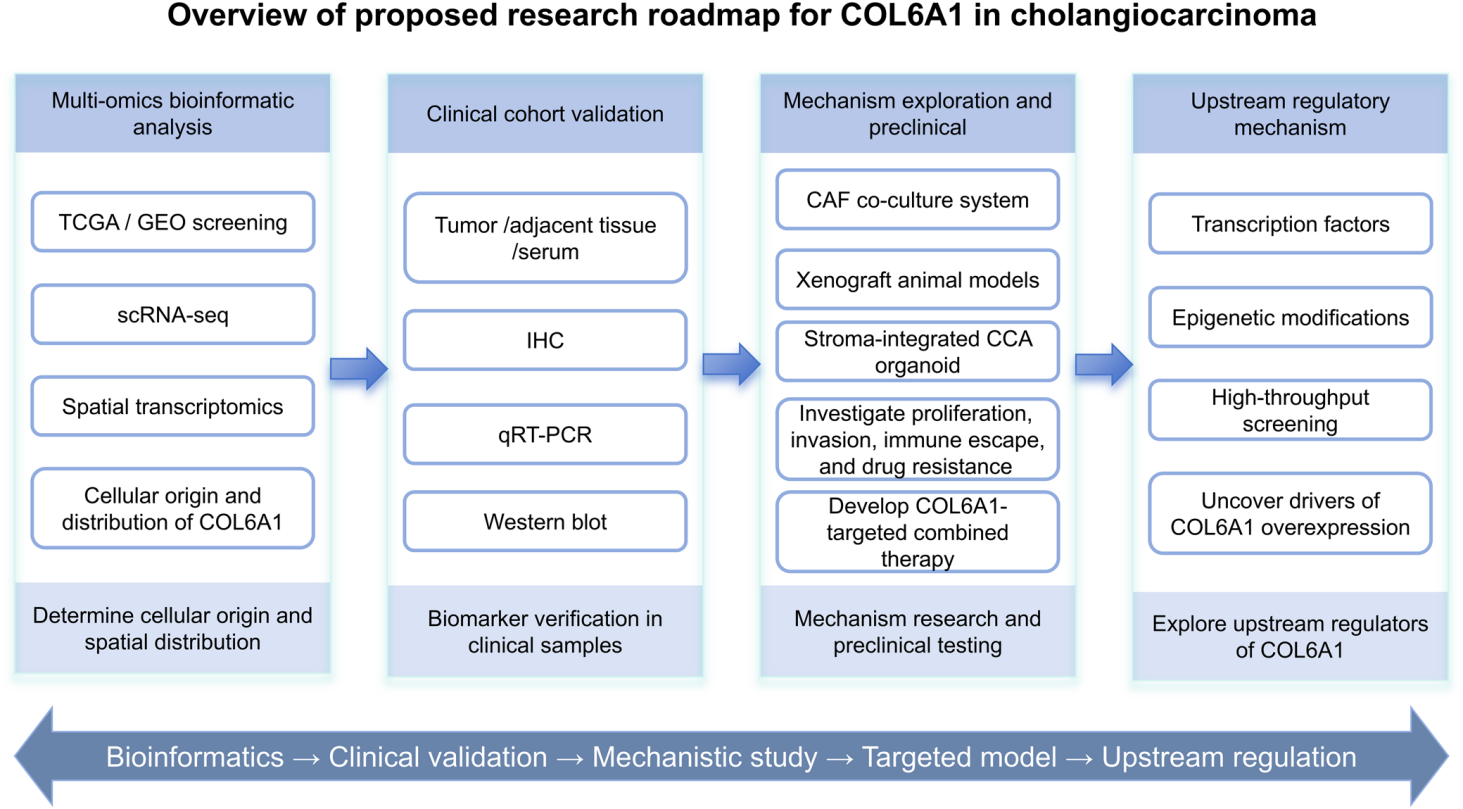
Overview of experimental platforms available for exploring COL6A1 in CCA research. This figure summarizes bioinformatic strategies, conventional in vitro and in vivo models, stroma‐containing CCA organoids, and detection approaches for clinical specimens, supporting subsequent mechanistic validation and translational studies.

First, multi‐omics strategies should be applied to explore the potential role of COL6A1 in CCA. Public transcriptomic datasets, including TCGA and GEO datasets, can be used for preliminary screening. Single‐cell RNA sequencing and spatial transcriptomics enable the precise identification of the cellular origin of stromal COL6A1 and characterization of its spatial distribution in the TME, providing systematic bioinformatic evidence and preliminary mechanistic clues for subsequent experimental validation. Second, large‐scale clinical trials have been conducted to collect tumor tissues, adjacent tissues, and serum samples from patients with CCA. Using immunohistochemistry, qRT‐PCR, and western blotting, we analyzed COL6A1 expression levels, clarified its pathological characteristics in CCA, and evaluated its correlation with clinical parameters (tumor stage, degree of differentiation, and metastatic status) and prognosis. This validated its potential as a diagnostic and prognostic biomarker. Third, we investigated the molecular mechanisms of COL6A1 using CCA cell lines, CAF coculture models, and animal tumor transplantation models. Our goal was to identify the specific molecular mechanisms that regulate COL6A1 in tumor proliferation, invasion, metastasis, immune evasion, and drug resistance. This includes pinpointing key downstream signaling molecules and their interactions to reveal the COL6A1–mesenchymal cells–tumor cell regulatory network. Fourth, we plan to develop more targeted therapies. Given the critical role of COL6A1 in pancreatic cancer metastasis, we aim to design and screen inhibitors, antibodies, and other targeted drugs. These will be validated in cell and animal models, and combined treatment strategies will be explored to provide an experimental foundation for clinical translation. Fifth, we aim to develop a precise research model by establishing a CCA organoid model with multiple stromal components that more accurately replicates the TME and provides a reliable platform for studying COL6A1 functions and drug screening. Sixth, we will investigate the regulatory mechanisms of COL6A1, focusing on upstream regulatory factors, such as transcription factors and epigenetic modifications, that drive its high expression in CCA, thereby establishing a solid theoretical foundation for comprehensively elucidating its role in this disease.

## 8 Conclusion

COL6A1, a crucial functional protein in the ECM, plays a pivotal role in the progression of various malignant tumors. It participates in the proliferation, invasion, and metastasis of tumor cells and significantly influences the regulation of the tumor immune microenvironment and the development of drug resistance. However, the role of COL6A1 in CCA remains relatively unexplored. Multiple challenges, including unclear mechanisms of action, incomplete research models, and lack of clinical translation studies, exist in COL6A1 research. However, this indicates a vast potential for future research. With continuous advancements in science, technology, and innovative research methods, efforts such as large‐scale clinical studies, in‐depth exploration of mechanisms, development of targeted therapies, precise research models, and investigation of COL6A1 regulatory mechanisms may gradually clarify its role in CCA. This will enhance our understanding of the pathogenic mechanisms, provide new diagnostic approaches and therapeutic targets, improve patient outcomes and quality of life, and improve research in this field.

## Author Contributions

**Weibin Zhang:** conceptualization, methodology, project administration, writing – review and editing, writing – original draft, funding acquisition. **Chongshi Gao:** writing – original draft, conceptualization, methodology. **Chongzhou Lan:** conceptualization, methodology, writing – original draft. **Jia Dong:** investigation, writing – original draft. **Boxing Xu:** investigation, writing – original draft. **Yingmei Li:** investigation, writing – original draft. **Jin Dai:** validation. **Baitong Che:** writing – original draft. **Cheng Li:** validation, writing – original draft. **Gang Dong:** validation, writing – original draft. **Yunfei Wu:** investigation, writing – original draft. **Bo Li:** investigation, validation, writing – original draft. **Cheng Yang:** writing – original draft, validation. **Lingmei Kong:** conceptualization, methodology, writing – original draft, writing – review and editing, project administration.

## Funding

This work was supported by the Natural Science Foundation of Liaoning Province (No. 2025‐MS‐371).

## Conflicts of Interest

The authors declare no conflicts of interest.

## Data Availability

The authors have nothing to report.
